# Radical Prostatectomy Survivorship: What Are We Really Asking?

**DOI:** 10.7759/cureus.72744

**Published:** 2024-10-31

**Authors:** Christopher Merrett, Arthur Yim, Xuan Rui Sean Ong, Benjamin Silagy, Abdullah Al-Khanaty, Deborah Stokes, Kate Slade, Gideon Blecher

**Affiliations:** 1 Urology, Monash Health, Melbourne, AUS; 2 Urology, Alfred Health, Melbourne, AUS; 3 Urology, Monash University, Melbourne, AUS

**Keywords:** erectile dysfunction, prostate cancer, prostatectomy, sexual dysfunction, survivorship

## Abstract

Introduction

Urinary incontinence, sexual dysfunction, and bowel dysfunction are well-recognized cancer survivorship outcomes affected by radical prostatectomy (RP) in the treatment of prostate cancer (PCa).The aim of this study was to audit the thoroughness of outpatient discussions and documentation of survivorship outcomes for patients who have undergone RP.

Methods

This was a retrospective audit of all 253 radical prostatectomies conducted at two tertiary-level Australian institutions (Monash Medical Centre and Alfred Hospital), over a five-year period between 2014 and 2018. Electronic medical records were reviewed across five time points: preoperative and then six weeks, three months, six months, and 12 months post-RP.The main outcomes measured were rates of documented discussions of urinary incontinence, sexual dysfunction, and bowel dysfunction.

Results

At the initial six-week postoperative review, there was an 86%, 47%, and 27% probability that urinary, sexual, and bowel functions were discussed and documented. When averaging across the four postoperative time points, from six weeks to 12 months, there was a 73%, 45%, and 14% respective rate of documented discussion for each survivorship outcome. Sexual and bowel function discussions were less frequently documented in men over 65 years of age compared with those under 65 years of age by 16% (p < 0.001) and 8% (p = 0.003), respectively.

Conclusion

Sexual and bowel dysfunction were less frequently discussed and documented in the follow-up of our cohort of RP patients compared with urinary continence outcomes.

## Introduction

Prostate cancer (PCa) is the most commonly diagnosed cancer in Australian men and accounted for 3,306 deaths in 2019 [1]. Australia has some of the highest estimated incidence rates of PCa at 86.4 per 100,000 men compared to the estimated worldwide incidence of 29.3 per 100,000 men [2]. While low-risk PCa is frequently managed with active surveillance, in order to delay the morbidity associated with curative treatments [3], radical prostatectomy (RP) has remained a common treatment modality for localized high-risk PCa, potentially as a part of a multi-modal treatment approach [4]. RP techniques have been developed from open, laparoscopic, and robot-assisted laparoscopic approaches. While there have been some differences in measured outcomes between these [5], there remains to be consensus on the ideal surgical approach. Despite this debate, there are well-recognized potential morbidities associated with each of the techniques [6,7]. The morbidity of any surgical therapy needs to be considered at all stages: preoperatively, intraoperatively, and in the postoperative setting.

While oncological outcomes maintain high importance to both clinicians and their patients, there continues to be a focus on patient survivorship, i.e., the "health and life of a person with a history of cancer" [8]. Despite the significant impact of adverse effects of cancer treatments and clinicians' good intentions to manage the patients holistically, several potential barriers may exist to have such detailed discussions. Some of these barriers include but are not limited to healthcare providers' unawareness of survivorship issues [9,10], the limited capacity of busy public clinics, financial constraints to provide relevant treatments, and inadequate communication between different healthcare providers [11]. This study was performed to ascertain the frequency with which clinical staff discuss and document patient’s survivorship outcomes following RP. The specific outcomes addressed include urinary continence and sexual and bowel dysfunction.

## Materials and methods

Following departmental ethical approval (Monash Health ad hoc 20744), a retrospective audit of all 253 patients who underwent RP was conducted at two tertiary-level public Australian institutions (Monash Medical Centre and Alfred Hospital) over a five-year period between 2014 and 2018. The records reviewed were originally paper-based and had been scanned into a browser-based medical record viewer. They were manually assessed by five separate researchers: three medical students, one surgical resident, and a surgical registrar. Patient interactions within each health service provider were separated into five time points: preoperatively and postoperative clinical reviews at six weeks, three months, six months, and 12 months. For each clinical encounter, we manually assessed whether the medical records or letters of communication clearly noted and documented discussions regarding urinary incontinence, sexual dysfunction, and bowel dysfunction.

No assessment was made on the suitability or completeness of any discussion, rather only that each outcome was discussed and documented. All interactions with health staff were considered including medical staff, nurses, and physiotherapists where appropriate. Patients were considered incontinent of urine if they expressed symptoms consistent with urinary incontinence requiring the use of any number of absorbent pads, condoms, or indwelling catheters (IDCs). Patients were considered to have sexual dysfunction if they had erectile dysfunction (ED) or any other sexual dysfunction that warranted discussions of treatment regardless of whether treatment was accepted by the patient or not. Any patients with bowel symptoms including urgency, diarrhea, anal incontinence, or constipation were considered to have bowel dysfunction. Statistical analysis to evaluate the association between age and survivorship outcomes was completed using Pearson's chi-square test with the null hypothesis rejected when the p-value was less than 0.05.

## Results

A total of 253 patients underwent RP over the five-year period between 2014 and 2018. There were 233 open RPs and 20 laparoscopic RPs performed. No robotic laparoscopic RPs were performed at either site. The median age at the time of operation was 64 years. Patients were discharged to other healthcare networks/private providers in 9% (n = 23) of six-week postoperative reviews and 25% (n = 64) at 12 months.

Urinary incontinence was discussed in 83% (n = 211) of patients preoperatively and its prevalence was 1% (n = 3). Preoperatively, there was a documented discussion of sexual dysfunction in 82% (n = 207) of patient files, revealing that 21% (n = 44) suffered from pre-existing sexual dysfunction, of which 9% (n = 4) were using treatments in the form of phosphodiesterase-5-inhibitors (PDE5i) or a penile prosthesis. There were no documented discussions of bowel dysfunction preoperatively across all 253 patient files (Table 1).

**Table 1:** Rates of documented discussion, prevalence when discussed, and treatments for each of the survivorship outcomes across all time points. IDC: indwelling catheter; AUS: artificial urinary catheter; PDE5i: phosphodiesterase type 5 inhibitor; ICI: intracavernosal injections; VED: vacuum erectile device

	Pre-op	Six weeks	Three months	Six months	12 months
Probability of documented discussion [n (%)]					
Urinary incontinence	211 (83)	218 (86)	175 (69)	179 (71)	170 (67)
Sexual dysfunction	207 (82)	119 (47)	103 (41)	117 (46)	117 (46)
Bowel dysfunction	0 (0)	68 (27)	45 (18)	18 (7.0)	12 (4.7)
Prevalence when discussed and documented [n (%)]					
Urinary incontinence	3 (1.4)	185 (85)	129 (74)	96 (54)	72 (42)
Sexual dysfunction	44 (21)	113 (95)	101 (98)	107 (92)	106 (91)
Bowel dysfunction	0 (0)	8 (12)	9 (20)	3 (17)	2 (17)
Treatment of urinary incontinence [n (%)]					
None	160 (100)	37 (19)	51 (32)	87 (55)	93 (62)
Pads	0 (0)	144 (74)	108 (67)	66 (42)	54 (36)
Medication	0 (0)	0 (0)	0 (0)	1 (0.6)	0 (0)
IDC	0 (0)	13 (6.7)	1 (0.6)	1 (0.6)	2 (1.3)
Sling	0 (0)	0 (0)	0 (0)	0 (0)	0 (0)
AUS	0 (0)	0 (0)	0 (0)	0 (0)	1 (0.7)
Treatment of sexual dysfunction [n (%)]					
None	230 (98)	127 (84)	78 (63)	73 (55)	72 (55)
PDE5i	3 (1.3)	22 (14)	39 (32)	40 (30)	35 (27)
ICI	0 (0)	0 (0)	2 (1.6)	14 (11)	19 (15)
VED	0 (0)	2 (1.3)	4 (3.3)	4 (3.0)	4 (3.1)
Prosthesis	1 (0.4)	0 (0)	0 (0)	0 (0)	3 (0.8)
Treatment of bowel dysfunction [n (%)]					
None	137 (100)	136 (99)	100 (98)	12 (80)	72 (97)
Bulking agents	0 (0)	1 (0.7)	0 (0)	1 (6.7)	0 (0)
Codeine	0 (0)	0 (0)	0 (0)	0 (0)	0 (0)
Referral	0 (0)	0 (0)	2 (2.0)	2 (13)	2 (2.7)

**Figure 1:**
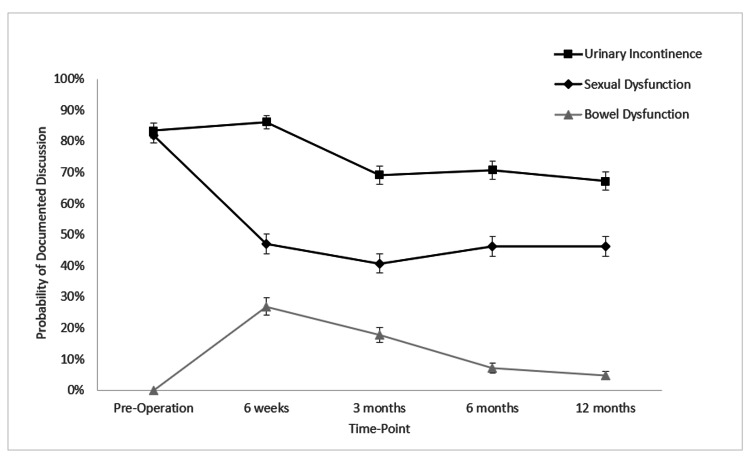
Rates of documented discussion for the survivorship outcomes across all time points. Error bars represent standard error.

Figure 1 displays the postoperative documented discussion of urinary incontinence of 86% (n = 218), 69% (n = 175), 71% (n = 179), and 67% (n = 170) at six weeks, three months, six months, and 12 months, respectively. This equated to rates of documented discussion of urinary function of 73% for any given time point. Postoperatively, the discussion of sexual dysfunction was 47% (n = 119), 41% (n = 103), 46% (n = 117), and 46% (n = 117) at six weeks, three months, six months, and 12 months, respectively. Overall, this equated to rates of documented discussion of sexual function of 45% for any given time point. Postoperatively, the discussion of bowel dysfunction was 27% (n = 68), 18% (n = 45), 7.0% (n = 18), and 5.0% (n = 12) at six weeks, three months, six months, and 12 months, respectively. This equated to rates of documented discussion of bowel function of 14% for any given time point.

When controlling for age, there was no significant difference between rates of documented discussion of urinary function (p = 0.482). In the setting of sexual dysfunction, men aged 65 and over were 16% less likely to have had a documented discussion relative to those men under 65 years of age (p < 0.001). For bowel function, men aged 65 and over were 8% less likely to have a document discussion compared to men under 65 years of age (p = 0.003).

**Figure 2:**
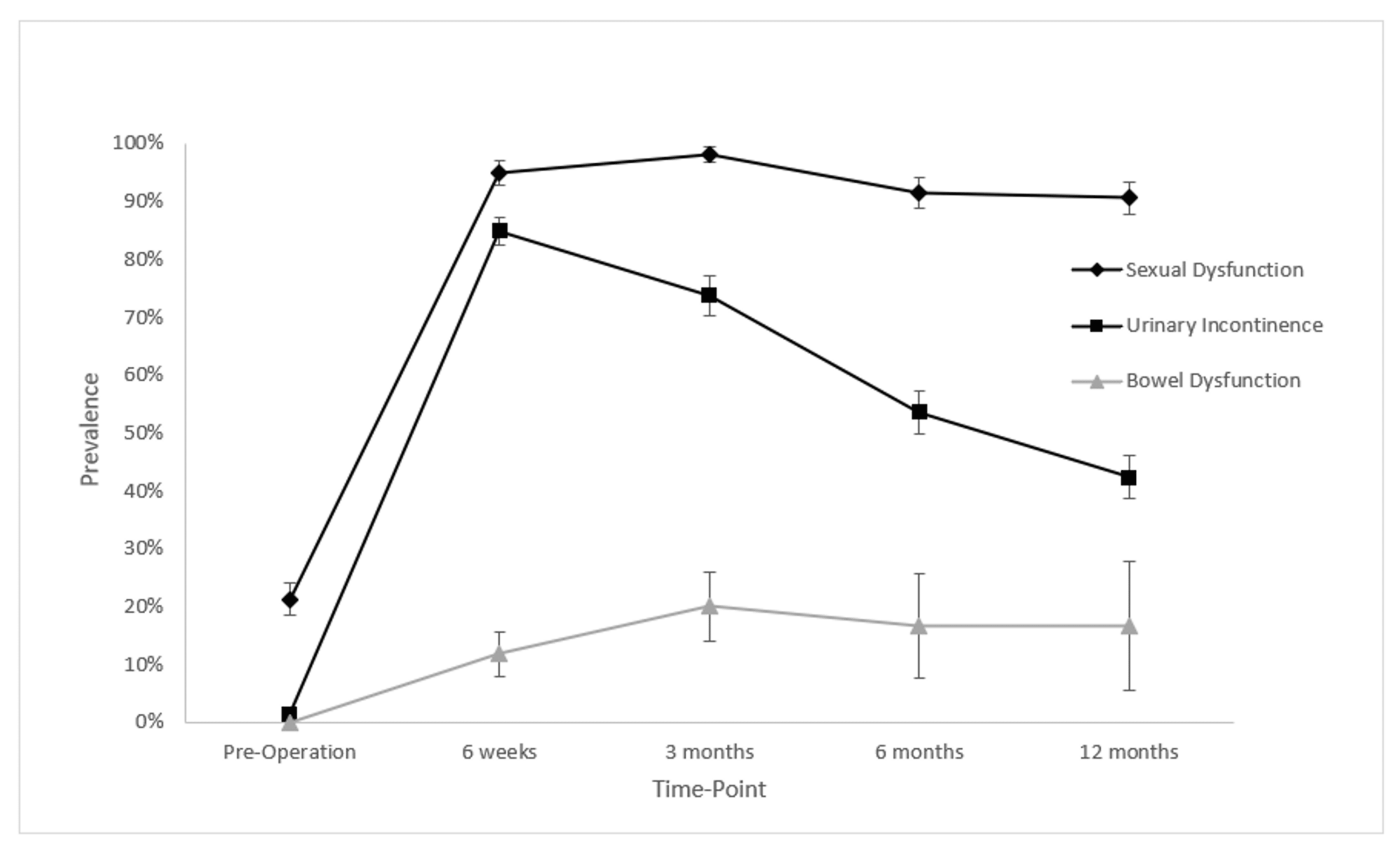
Prevalence of each survivorship outcome across all time points when documented discussions were made. Error bars represent standard error.

Figure 2 highlights the decreasing prevalence of urinary incontinence from 85% (n = 185) at six weeks to 42% (n = 72) at 12 months. The use of urinary pads was the most common choice of treatment, with 73% (n = 144) using them at six weeks and 35% (n = 54) still using them at 12 months. An AUS was the treatment of choice in one patient for 12 months. The prevalence of sexual dysfunction in our patient population decreased from the six-week follow-up at 95% (n = 113) through to the 12-month follow-up at 91% (n = 106). The commencement of pharmacological or mechanical therapies for the treatment of sexual dysfunction was seen to increase over time from 26% (n = 24) at six weeks to 57% (n = 61) at 12 months. By 12 months, 26% (n = 35) were using PDE5is, 15% (n = 19) using intracavernosal injections (ICIs), 3% (n = 4) using vacuum erectile devices (VED), and 1% (n = 3) undergoing surgical management with a penile prosthesis. The prevalence of bowel dysfunction was stable from 12% (n = 8) at six weeks to 17% (n = 2) at 12 months. There were a total of six referrals made to other medical specialties for the treatment of bowel dysfunction across the 12-month follow-up period.

## Discussion

Adverse effects are common post-RP. Urinary incontinence is the involuntary loss of urinary control. It is common with an estimated prevalence in the scientific literature of up to 80% post-RP [12]. First-line treatment for urinary incontinence post-RP involves pelvic floor exercises and the use of continence pads [13]. Artificial urinary sphincters (AUSs) and male slings are surgical procedures to treat those who fail pelvic floor physiotherapy. Although much less common with RP (reported prevalence of up to 17% [14,15]) compared to radiotherapy, bowel dysfunction may pose an issue, comprising of rectal urgency, loose bowel actions, or anal incontinence.

Sexual dysfunction may occur in up to 90% of men post-RP [16-18]. Apart from universal anejaculation, the most common variable symptom of sexual dysfunction in the post-RP patient is ED [19]. ED is the self-reported inadequate penile erectile function to complete desired sexual activity [20]. The pathophysiology of ED in the setting of an RP is likely the result of disruption to the erectile neurovascular bundles either from traction, thermal, or direct injuries contributing to denervation and ischemia possibly contributing to local fibrosis to the corpora cavernosa and dysfunction [21]. Sexual dysfunction is associated with significant emotional distress and relationship issues [22,23]. Postoperative management of ED is usually treated initially with oral PDE5is and mechanical vacuum erection devices (VEDs). Those men with PDE5i refractory ED often trial ICI. The end-stage treatment option involves surgical implantation of a penile prosthesis.

Despite the importance of addressing these adverse effects, there are several potential barriers to the identification and discussion of survivorship outcomes in the postoperative setting. They may be competing for discussion time among the oncological aspects of cancer survival. Patients initially are more likely to be focused on their oncological outcomes - the somewhat medium-term aspect of this audit of up to 12 months post-intervention may reflect this. It is important that healthcare workers are aware of this potential bias and not neglect sexual, urinary, and bowel survivorship outcomes that all affect their patient’s quality of life. The age of the patient is an important factor. In pediatric oncology, children reportedly typically have better follow-up care and monitoring systems in place, resulting in an overall superior identification and care of survivorship outcomes when compared to adults [8]. In our studied adult patient population, there was a statistically significant difference in older patients with men aged over 65 being less likely to have a documented discussion about their sexual and bowel function.

There was variation in the rates of documented discussions of the three survivorship outcomes, urinary continence, sexual, and bowel function. Urinary function appeared to be somewhat well addressed with rates of 73% that at each time point for each man they had a documented discussion. It has been reported that urinary incontinence possibly causes more distress when compared to other survivorship outcomes including sexual and bowel dysfunction [24]. There has also been evidence that initiating pelvic floor exercises in the preoperative setting may also lead to improved continence [25].

Sexual dysfunction appeared less likely to be discussed relative to urinary continence with a 45% probability of documented discussion for each given time point for each man. This was despite a high estimated prevalence of sexual dysfunction where there was a documented discussion, up to a maximum of 98% at the three-month time point. Patients may be unlikely to spontaneously discuss their sexual dysfunction, although there may exist a possible reluctance on the healthcare workers to prompt them. This is despite the body of research that links sexual dysfunction with depression and suicide [26]. It is also possible that healthcare workers may be having these discussions and neglecting to document when sexual function was normal.

Bowel dysfunction also appeared much less likely to be addressed relatively to urinary function with a 14% probability of a documented discussion at any given time point for any given man. In the literature, this adverse outcome is relatively rarer in this population of men compared to sexual and urinary dysfunction. This was reflected in our results, with up to 18% of men having reported bowel dysfunction at 12 months when a discussion was documented. Our results are consistent with previously published work by Sonn et al. 2013 who demonstrated under-reporting by physicians of various quality-of-life outcomes in the follow-up of PCa treatment, including that of urinary incontinence, sexual dysfunction, and bowel dysfunction [27].

A survivorship outcome that was not included in this audit was psychological stress. Whilst it remains a significant aspect of cancer patient care, we elected not to include it as there was great variability in its documentation. Neither of the studied institutions in this study routinely used a validated quality of life (QoL) questionnaire. Any future prospective study on this topic should consider including a cancer-specific validated QoL questionnaire such as the QLQ-C30.

This study was limited by its reliance on documentation to audit outcome measures. There may have been occasions where a discussion was had between the healthcare worker and the patient that was not documented. Ideally, the use of validated reporting tools and patient-reported outcome measures could enhance prospective data collection and provide a robust method for health services to improve their model of care. Institutions, such as the Memorial Sloan Kettering Cancer Centre, report regular routine use of validated questionnaires in the postoperative care of their patients who have undergone RP [28]. Another potential method of improving survivorship discussion rates could be dedicated post-RP clinics with appropriate allied health staff including physiotherapists, specialist nursing staff, and psychosexual counselors.

## Conclusions

In the postoperative RP setting, urinary incontinence seems well addressed, whilst other outcomes such as sexual and bowel dysfunction are less frequently discussed. The increasing age of the patient appears to affect the likelihood of discussions around sexual and bowel dysfunction survivorship outcomes. These outcomes are important to patients and their oncological journey. To optimize the care of our patients in the postoperative setting, we should be actively discussing these issues and liaising clearly with their primary care providers.
